# Research on Optical Fiber Sensor Based on Underwater Deformation Measurement

**DOI:** 10.3390/s19051115

**Published:** 2019-03-05

**Authors:** Jiawang Chen, Chen Cao, Yue Huang, Yonglei Zhang, Yongqiang Ge

**Affiliations:** Ocean College, Zhejiang University, Zhoushan 316021, China; arwang@zju.edu.cn (J.C.); 21834170@zju.edu.cn (Y.H.); yonglei@zju.edu.cn (Y.Z.); Ge_yongqiang@zju.edu.cn (Y.G.)

**Keywords:** underwater deformation, sensitive region, optical power loss, fiber curvature sensor, measurement range

## Abstract

With the increase in the scale and complexity of underwater engineering, safety problems caused by underwater deformation have become increasingly prominent. Although the intensity fiber curvature sensor can be used for curvature monitoring on the ground, its sensing mechanism is still under investigation. This paper establishes the mathematical model of optical power relative loss and bending radius during deformation of the fiber sensitive region and uses the optical power meter to measure light intensity loss in the sensitive region, which verifies the correctness of the model and reveals the sensing mechanism of the intensity fiber curvature sensor, then optimizes the sensor signal conditioning circuit, applies the sensor to the single-point deformation curvature measurement, and analyzes its measurement error and accuracy. It is proved that the linear measurement range of the sensor is improved when compared with existing similar products.

## 1. Introduction

With the vigorous development of the marine industry in China, the quantity and scale of underwater engineering have been increasing, and underwater deformation detection is imperative [1,2]. For example, the stability of the subsea gas hydrate structure is so susceptible to temperature and pressure changes that its exploitation may result in drastic changes in the seabed topography, destroying subsea engineering facilities such as surrounding oil pipelines. The underwater topographic survey determines the plane coordinates and depth of the underwater topographic point [3]. With the development of underwater acoustic measurement, GPS positioning and computer technology [4], underwater topographic surveys have gone into a new period from traditional optical positioning, single-beam sounding, manual data processing to make use of GPS positioning and multiple sounding depths with automated data processing and diversification of results [5]. Compared with the commonly used single beam, multi-beam, side-scan sonar, GPS [6,7], MEMS and other measurement methods, the intensity type fiber curvature sensor not only has better characteristics than traditional sensor technology, such as large broadband information capacity, long-distance transmission, strong anti-electromagnetic interference capability, strong security and confidentiality [8], but also possesses the advantages of simple structure, low cost, wide measuring range and easy implementation. At present, the monitoring technology of domestic underwater engineering is still in its infancy [9,10], so it is of great significance to apply fiber-optic sensors to the measurement of underwater deformation.

Although the fiber curvature sensor has been widely used in the field of aquatic intelligent health monitoring, the sensing mechanism is still being explored, including the assumptions of Lee Danisch and optical loss, KSC Kuang and the region and R Philip-Chandy and the sensitive region shape [11]. However, most of these explanations lack the support of theoretical and experimental results. They are in the guessing stage which cannot accurately and effectively explain the working mechanism of the intensity-type fiber curvature sensor. Therefore, it is necessary to establish a mathematical model to quantitatively analyze the sensor to reveal the sensing mechanism of this type of sensor.

In recent years, domestic scholars have begun to study this type of sensor. Fu Yili, Liu Renqiang, Di Haiting and others have done some research on the principle characteristics and application of intensity-modulated fiber-optic sensors. Liu Renqiang developed a buried fiber curvature sensor based on the sensitive region of the long-length fiber. It can be used for shape detection of intelligent structures such as bridges [12]. Di Haiting developed a new quasi-distributed intelligent sandwich sensing system based on the sensitive region for the sawtooth fiber curvature sensor, which can be used to monitor the bending deformation of composite structures [13]. However, the above sensor measurement systems are complex and the measurement range is narrow. Therefore, this paper improves and optimizes the sensor measurement system so that it can be better used for underwater deformation measurement.

## 2. Optical Power Loss in Bending of the Fiber Sensitive Region

**Figure 1:**
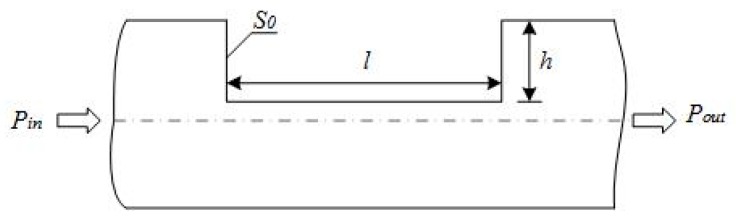
Shape of the fiber sensitive region.

Traditional fiber-optic sensors which use fiber macro-bend loss to measure curvature have no surface treatment [14], so sensitivity is very low and the bending direction cannot be distinguished, which is difficult to apply in practical engineering. As shown in Figure 1, a strip-shaped light leakage region of length l and depth h is processed on the surface of the optical fiber to increase the amount of light leakage when the optical fiber is bent, thereby increasing the sensitivity of the optical fiber to bending. Generally, when the positive direction of the fiber sensitive region (the sensitive region is located on the convex side of the curved fiber) is bent and the negative (the sensitive region is located on the concave side of the curved fiber sensor) is bent, the amount of light leakage is different; when the positive direction is bent, the light leakage is larger; while the light is bent in the negative direction, the light leakage is less [15]. This paper will use the theory of light to establish a mathematical model between the relative loss of optical power and the bending radius of the fiber sensitive region to reveal the working mechanism of the intensity modulated fiber sensor [16,17]. Since the measurement range of the fiber curvature sensor is relatively wide, the plastic fiber SH-4001 is used.

### 2.1. Transmission Power of the Optical Fiber Sensitive Region during Bending

#### 2.1.1. Positive Bending

For the light propagating in the core, part of the light reaches the sensitive region and leaks out of the core, and another part of the light continues to propagate along the fiber. The optical power of the micro-cell $ds_{0}$ on the $s_{0}$ plane leaking to the outside of the core at the dΩ angle is $dp_{0}$, and in the positive bending state (the sensitive region is on the convex side), the optical power $p_{0}$ leaking from the surface $s_{0}$ can be expressed as [11]:

(1) $$\begin{array}{l} P_{0}=\iint_{S_{0}}dS\int_{0}^{2\pi }d\theta_{\varphi }\int_{0}^{\theta_{b}}I_{0}\sin\theta_{0}\cos\theta_{0}d\theta_{0}\\ =2\pi I_{0}\int_{a-h}^{a}2\sqrt{a^{2}-h_{1}^{2}}dh_{1}\int_{0}^{\theta_{b}}\sin\theta_{0}\cos\theta_{0}d\theta_{0}\\ =2\pi I_{0}\int_{a-h}^{a}J(h_{1})dh_{1} \end{array}$$

where h is the depth of the sensitive region and when the light passes through the surface $s_{0}$, the optical power ratio $\alpha_{{}_{0}}$ of the leakage is:

(2) $$\alpha_{0}=P_{0}/P_{b}=\int_{a-h}^{a}J(h_{1})dh_{1}/\int_{-a}^{a}J(h_{1})dh_{1},$$

Assuming that the depth h of the processed fiber sensitive region is uniform and the light in the core leaks out of the core at the sensitive region, it is completely absorbed by the medium outside the core and is no longer returned to the core. According to the assumption, the fiber sensitive region of length l is regarded as N $s_{0}$ planes, and the leakage ratio of optical power of each surface is $\alpha_{{}_{0}}$, so the relationship between the output optical power and the bending radius R of the fiber and the depth h of the sensitive region, that is, the mathematical model of the positive direction bending resulting in optical power loss is:

(3) $$P_{out}=P_{b}{\left(1-\alpha_{{}_{0}}\right)}^{N},$$

The relative loss of optical power in the sensitive region can be expressed as:

(4) $$\delta P=(P_{b}-P_{out})/P_{b},$$

The relative loss of optical power in the sensitive region is obtained when the optical fiber sensor is in a positive bending state.

(5) $$\delta P=1-{\left(1-\alpha_{{}_{0}}\right)}^{N},$$

#### 2.1.2. Negative Bending

Similar to the case of positive bending, in the negative bending state (the sensitive region is on the concave side), the optical power leakage from the surface $s_{0}$ can be expressed as [11]:

(6) $$\begin{array}{l} P_{0}{}^{\prime }=\iint_{S_{0}}dS\int_{0}^{2\pi }d\theta_{\varphi }\int_{0}^{\theta_{b}}I_{0}\sin\theta_{0}\cos\theta_{0}d\theta_{0}\\ =2\pi I_{0}\int_{-a}^{-a+h}2\sqrt{a^{2}-h_{1}^{2}}dh_{1}\int_{0}^{\theta_{b}}\sin\theta_{0}\cos\theta_{0}d\theta_{0}\\ =2\pi I_{0}\int_{-a}^{-a+h}J(h_{1})dh_{1} \end{array}$$

Then the optical power factor of the leakage at the surface is:

(7) $$\alpha_{0}{}^{\prime }=P_{0}{}^{\prime }/P_{b}=\int_{-a}^{-a+h}J(h_{1})dh_{1}/\int_{-a}^{a}J(h_{1})dh_{1},$$

Similarly, in the negative bending state of the fiber sensor, the relative loss of optical power in the sensitive region can be expressed as:

(8) $$\delta P^{\prime }=1-{\left(1-\alpha_{{}_{0}}{}^{\prime }\right)}^{N},$$

**Figure 2:**
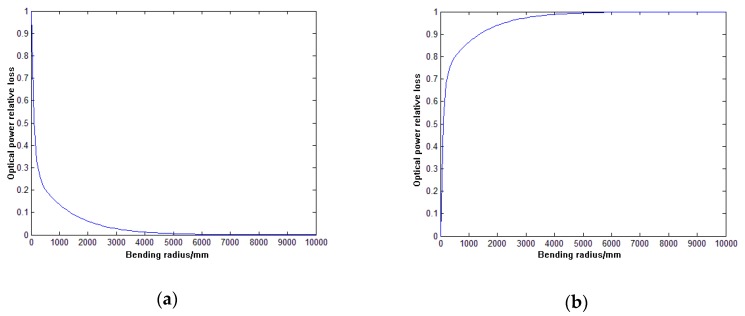
(**a**) relationship between positive bending relative optical power loss and bending radius; (**b**) relationship between negative optical bending relative optical power loss and bending radius.

According to the formula (5) and the formula (8), the relationship between the relative loss of the optical power in the sensitive region and the bending radius is plotted. As shown in Figure 2, for the long sensitive region, the relative loss of optical power decreases with the increase of the bending radius in the positive bending; while the relative loss of the optical power increases when the bending radius in the negative bending increases.

### 2.2. Transmission Power of the Optical Fiber Sensitive Region during Bending

**Figure 3:**
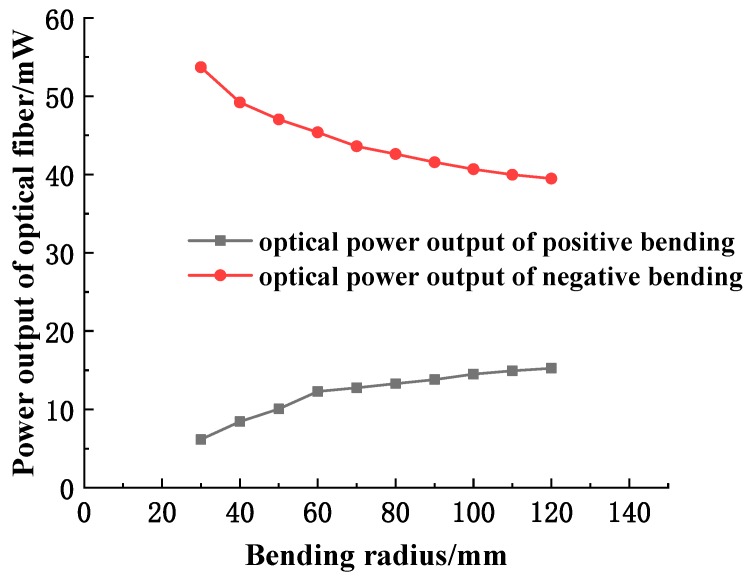
The relation curve of output light intensity and bending radius of fiber.

According to the directional nature of the bending, a certain degree of deformation is performed on the sensitive regions of the optical fiber, respectively. The optical power meter THORLABS PM200 is used to measure the output intensity of the fiber in different bending radii of the fiber sensitive region. Finally, the output light intensity and bending of the fiber are drawn. The curve relationship of the radius is shown in Figure 3.

According to the curve, the relationship between the output power of the fiber and the bending radius can be known as follows: When the sensitive region of the fiber is bent positively, the output optical power of the fiber gradually increases with the increase of the bending radius; when bending is negative, the opposite is true.

**Figure 4:**
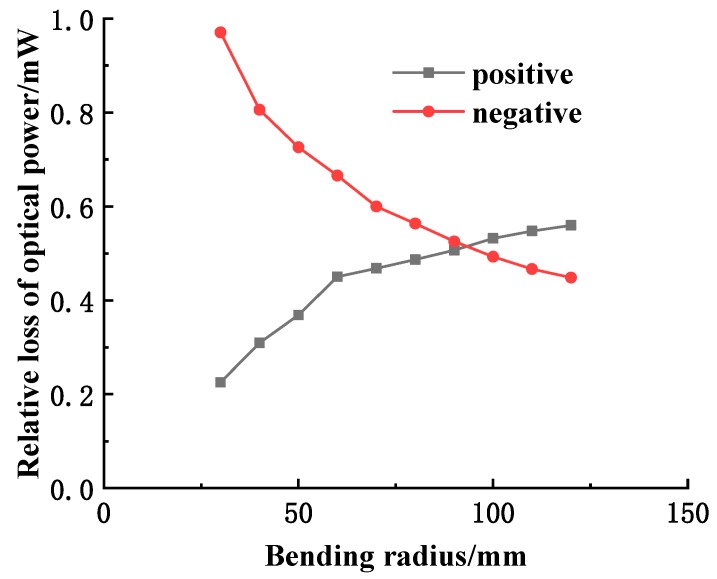
The relation curve between the relative loss of optical power and bending radius.

Being aware of the output intensity of the fiber when the fiber sensitive region is not bent, according to the measured output optical power of the fiber under different bending radii, the relationship between the relative loss of the optical power of the fiber curvature sensor and the bending radius of the fiber would be obtained. As shown in Figure 4, when the bending radius is large (≥120 mm), since the output voltage of the fiber curvature sensor has no obvious relationship with the bending radius, the second half of the curve cannot be proved in Figure 2. However, within the linear measurement range of the sensor from 20–120 mm, the measured curve is basically consistent with the simulation results in the mathematical model, indicating that the established mathematical model is effective.

## 3. Sensor Signal Conditioning Circuit

**Figure 5:**
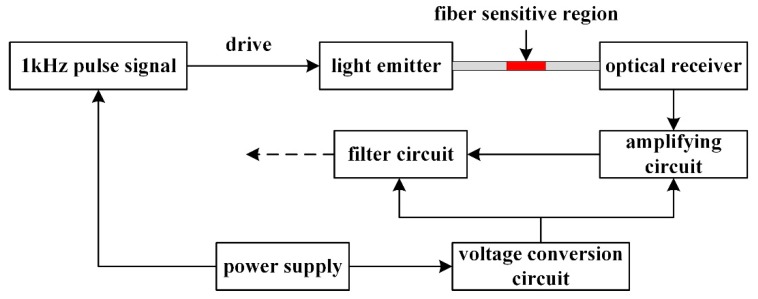
Sensor signal conditioning flow chart.

The sensor conditioning circuit mainly includes functional module circuits such as amplification, filter, voltage conversion and so on, which can recognize the weak signal change of the sensor output. As shown in Figure 5, when the pulse signal drives the light emitter and the red light emitted by the light emitter is transmitted through the optical fiber, the bending information of the sensitive region of the fiber is converted into the voltage signal which is the output of the light receiver. Then the signal is amplified and filtered, finally being obtained by the microcontroller.

### 3.1. Amplifying Circuit

The input signal of the sensor is the bending information of the sensitive region of the fiber, and the output signal is a weak voltage signal. Generally, it is within 50 mV, and with the positive and negative bending of the sensitive region of the fiber, the dynamic range of the signal is wide, which, in the meantime, is easily affected by the working environment. Therefore, the input at the next stage must have large common-mode interference. In order to reduce the interference, the instrumentation amplifier AD620 is selected in the amplifier circuit for amplification. Compared to general-purpose amplifiers, AD620 has the advantages of the small operating current, high common-mode rejection ratio, low offset and drift, low noise and high closed-loop gain stability [18,19].

**Figure 6:**
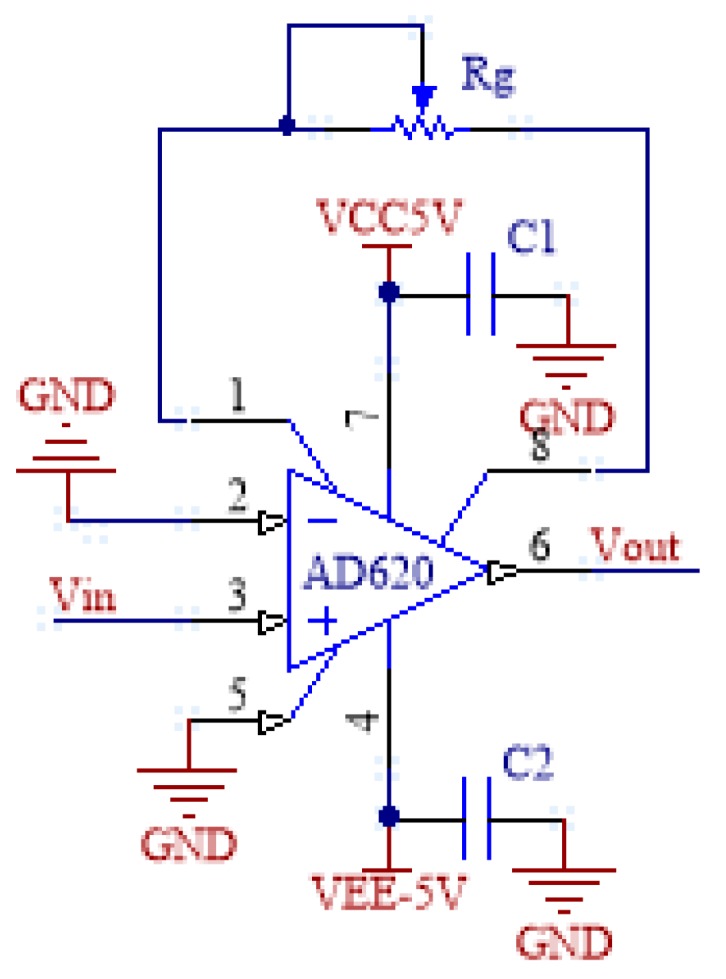
AD620 amplifier circuit.

As shown in Figure 6, the amplifying circuit is a single-ended input: the inverting input of the AD620 is directly grounded, and the non-inverting input is connected to the sensor output signal. Meanwhile, this circuit can also play an important role in reducing common mode noise. Because the sensor output is also a single-ended signal and the other end is actually shared with the entire circuit system, it can be used as a double-ended input. In addition, a variable resistor $R_{g}$ is connected across the circuit pins 1 and 8 to facilitate adjustment of the amplification factor G. Both dual power supply inputs are connected to the ground through a capacitor to ensure the stability of the supply voltage.

### 3.2. Filter Circuit

The pulse frequency of the driving photodiode is 1 kHz, so the center frequency of the sensor output signal is about 1 kHz. The output signal of the sensor can be modulated by band-pass filtering. OP07 is chosen as the main chip [20]. The chip’s high accuracy and extremely low input offset voltage eliminate the need for additional zeroing in many applications [21].

**Figure 7:**
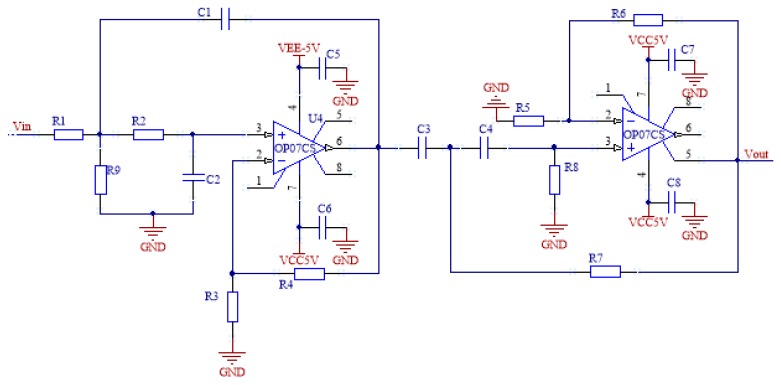
Band-pass filter circuit.

According to relevant knowledge of the analog circuit [22], the band-pass filter circuit can generally be formed by a low pass filter circuit and a high pass filter circuit connected in series. In order to reduce the output voltage at a faster rate outside the passband, improving the ability of the band-pass filter to remove noise, the second-order low-pass and second-order high-pass filter circuits are connected in series to form a fourth-order band-pass filter. As shown in Figure 7, the amplitude-frequency characteristic of the circuit is narrow, and the gain of the passband is 0 dB. At the frequency of 650 Hz or 1350 Hz, the amplitude attenuation is about 3 dB.

### 3.3. Summing Circuit

**Figure 8:**
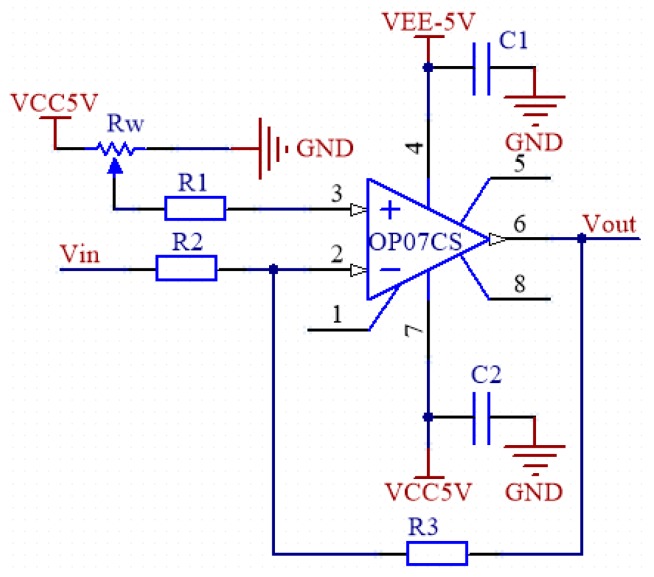
Band-pass filter circuit.

The micro signal output by the sensor has been amplified and filtered, and the final output signal is supposed to be a sine wave voltage signal [23]. If the sine wave signal is directly connected to the IO pin of the A/D input of the microcontroller, only the positive voltage in the range of 0–3.3 V could be measured, while the negative voltage would directly return to 0. Therefore, it is also necessary to carry out bias processing on the signal to raise the whole sine wave signal above the 0 level and ensure that the maximum amplitude of the signal is below 3.3 V. According to the principle of voltage divider, the designed circuit is shown in Figure 8. By adjusting the resistance of the slide rheostat to change the value of $V_{k}$, the sinusoidal signal can be moved up and down as a whole, and the sinusoidal signal waveform can be controlled between 0–3.3 V.

## 4. Sensor Characteristics Analysis

### 4.1. Linear Measurement Range Analysis

#### 4.1.1. A Sensitive Region

**Figure 9:**
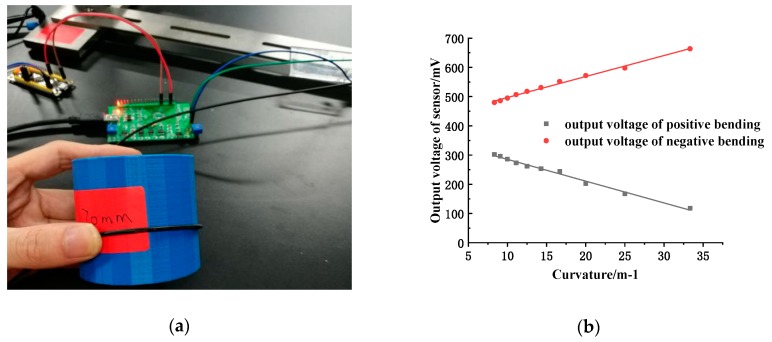
(**a**) bending on a cylinder; (**b**) linear fitting of positive and negative bending output voltage on a cylinder.

As shown in Figure 9a, the sensitive region of the fiber is sequentially pressed against the surface of a series of cylinders with a radius of 10 mm, 20 mm, …, 120 mm for positive and negative bending. According to the output voltage of the sensor, the relationship curve between the output voltage of the sensor and the bending curvature can be obtained through linear fitting within a certain range, as shown in Figure 9b:

The linear equations of the sensor in positive and negative bending are obtained by linear fitting:

(9) $$U_{postive}=359.81715-7.45486C,$$

(10) $$U_{negative}=425.09514+7.18588C,$$

It can be seen from the above graph that the two fitting lines when the fiber is positively and negatively bent are basically symmetrical about the voltage value of 400 mV (that is, the output voltage value of the sensor when the fiber sensitive region is not bent). The measurement range of the fiber curvature sensor is defined as 8.33 to 33.3 m^−1^. Within this range, the output voltage of the sensor is linearly related to the curvature.

#### 4.1.2. Two Sensitive Regions

**Figure 10:**
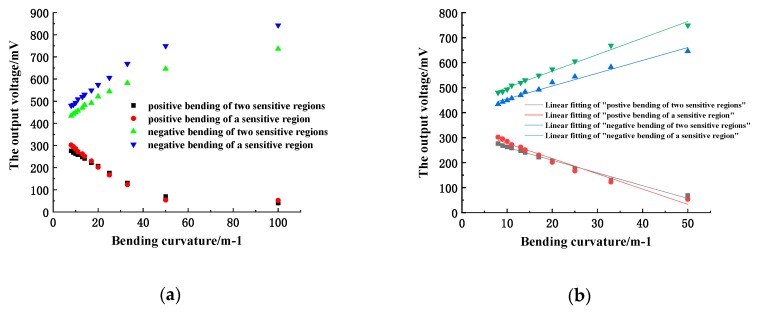
(**a**) relationship between output voltage of two sensitive regions and bending curvature; (**b**) linear fitting of two sensitive regions.

Two sensitive regions of the same length are processed on a fiber with a length of 1 m and a gap of 250 mm. When there is only one sensitive region, the voltage when the fiber is not bent is 400 mV, while after the second sensitive region is processed, the output voltage drops to 370 mV. The first sensitive region on the optical fiber was bent on a series of cylindrical cylinders, measuring the output voltage before and after processing the second sensitive region, then the relationship curve between the output voltage of the sensor and the bending curvature could be obtained, as shown in Figure 10a. The curve is fitted with a straight line within the range of linear measurement, as shown in Figure 10b.

The curve shows that the linear measurement range of the sensor remains substantially unchanged after the second sensitive region is added. However, the output voltage will decrease when the fiber is bent, that is, the optical power loss will increase. According to the fitted line, it can be seen that after increasing the number of sensitive regions, the sensitivity of the sensor will decrease when bending positive or negative. Therefore, once the sensor’s measuring sensitivity is guaranteed, multiple sensitive regions can be arranged on one optical fiber to realize the curvature measurement of multiple points.

### 4.2. Underwater Measurement Accuracy Analysis

#### 4.2.1. Underwater Simple Beam Measurement

On land, the sensor can detect bending curvature within a certain range. Before applying it underwater, it is necessary to verify whether the sensor still meets some rules of underwater. Due to the limited experimental conditions, an experimental method similar to simply supported beams is used to roughly examine the characteristics of the sensor when it is used underwater.

**Figure 11:**
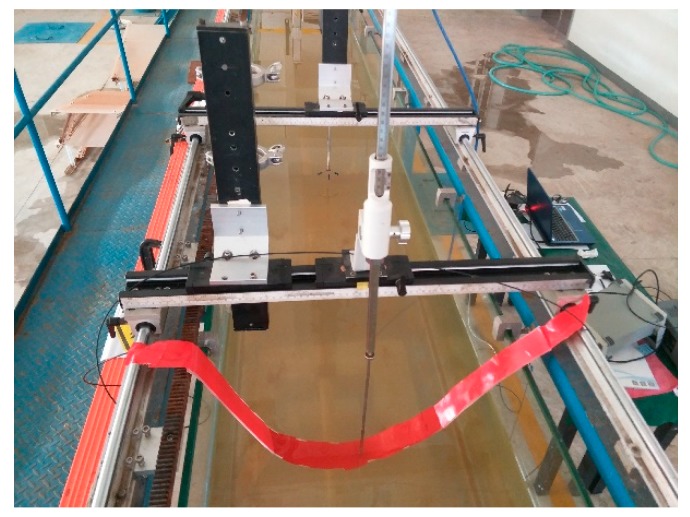
Underwater simply supported beam measurement experiment.

**Figure 12:**
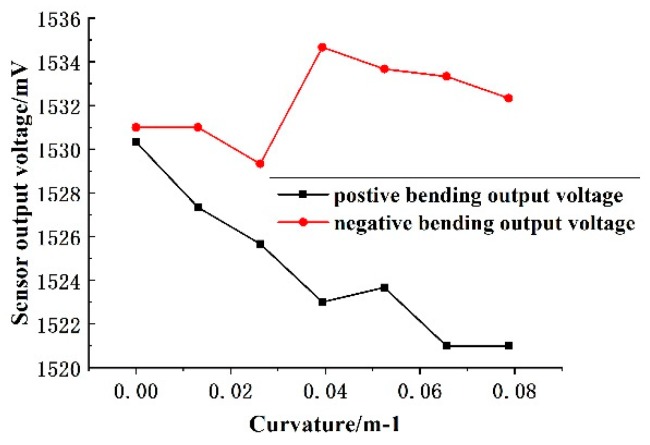
Output voltage versus curvature curve of underwater simply supported beam bending.

As shown in Figure 11, the fiber sandwich is placed on a 50 mm wide water tank, and then directly traversed the sensitive area of the fiber interlayer through the thimble with the scale mark. The output voltage value of the sensor is recorded every 5 cm; until the thimble moves and the distance reaches 35 cm, the fiber interlayer is turned 180 degrees, the negative bending is measured according to the above operation, and finally the relationship between the sensor output voltage and the curvature is plotted, as shown in Figure 12.

The curve in the figure shows: When the bending curvature of the fiber varies from 0–2.7 m^−1^, the overall change trend of the output voltage of the sensor is consistent with the theory of power loss in the sensitive area of the fiber, that is, negative (positive) bending, as the bending curvature increases, the output voltage increases (decreases). However, from a partial perspective, there is no significant relationship between the output voltage of the sensor and the bending curvature of the fiber. Therefore, it can be concluded that the sensor is not suitable for measuring deformations with a curvature range of 0–2.7 m^−1^; the power loss principle of the fiber sensitive area is still applicable underwater.

#### 4.2.2. Underwater Dynamic Curvature Measurement

**Figure 13:**
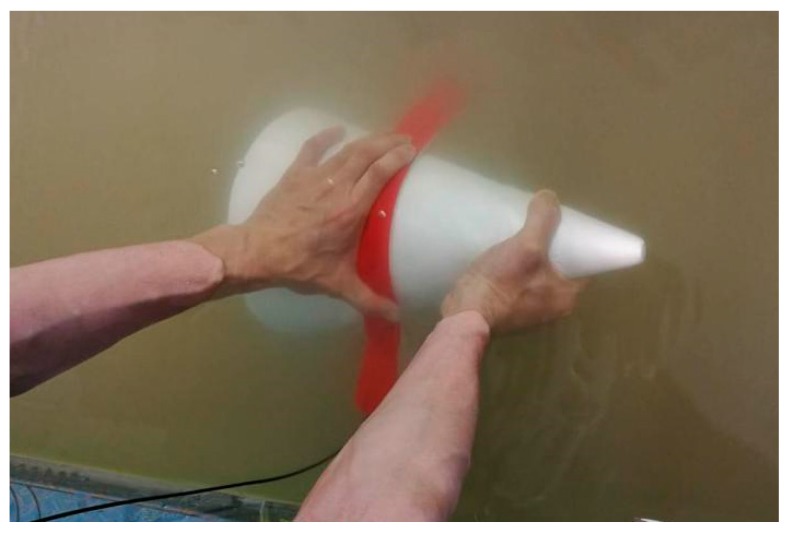
Measuring the curvature of the cone.

As shown in Figure 13, the optical fiber interlayer, is attached to the surface of a core with a bottom diameter of 30 cm and a height of 35 cm (taper of 0.86) underwater, moving slowly upward from the side of the cone near the bottom (the sensitive regions of the fiber are always kept close to the side of the cone in the process of moving), and the single-chip microcomputer is used to collect the real-time output voltage of the sensor.

**Figure 14:**
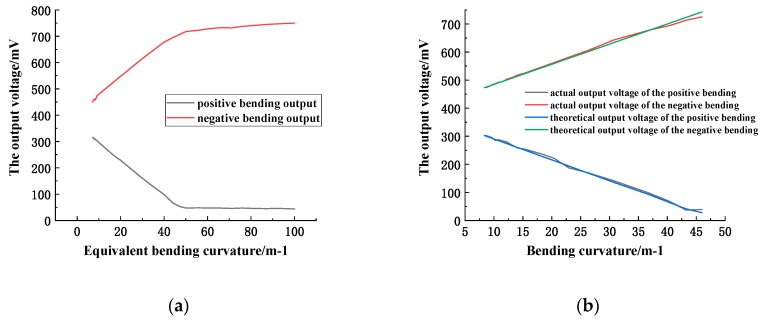
(**a**) relationship between output voltage and equivalent bending curvature; (**b**) relationship between the actual measured and theoretical output voltage and the bending curvature.

The relationship between the sensor output voltage and the equivalent bending curvature is plotted according to the measured data (the equivalent bending radius is the cross section radius parallel to the base of the cone at the contact point between the sensitive region and the cone’s side, which is proportional to the actual bending radius, and the ratio coefficient is 0.92), as shown in Figure 14a.

It can be seen from the above curve that when the equivalent bending curvature is in the range of 10–50 m^−1^ (the actual bending radius is about 30–120 mm), the output voltage is also substantially linear with the equivalent bending curvature. Within this range, compare the actual output voltage to the theoretical voltage (calculated by using the linear measurement equation of the sensor) and a curve varying with the curvature of a curve are plotted. As shown in Figure 14b, the relationship between the actual, theoretical output voltage and the curvature of curvature is substantially coincident, indicating that the measuring range of the sensor is also suitable for its underwater measurement.

#### 4.2.3. Measurement of Underwater Static Curvature

**Figure 15:**
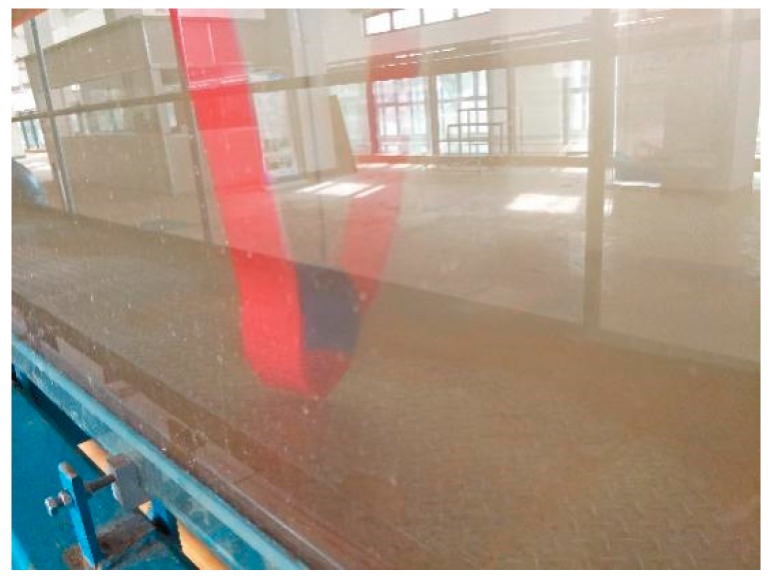
Measuring the curvature of a cylinder.

As shown in Figure 15, the standard cylinder is regarded as the underwater deformation with known curvature. The sensitive region of the fiber is sequentially attached to the cylindrical surface with a radius of 120 mm, 100 mm, …, 40 mm, 20 mm. The sensor’s output voltage values of each time are correspondingly substituted into Equations (11) and (12) to obtain the measured curvature.

According to the Table 1 and the calculation, when the positive and negative bending radius of the fiber is 60 mm, the deviation between the measured curvature and the theoretical curvature is the maximum, but the deviation is different. $\Delta C_{positive\;\max}=0.76\;m^{-1}$ and $\Delta C_{negative\max}=0.6\;m^{-1}$. Therefore, the measurement accuracy of the sensor in the positive and negative linear range can be obtained as follows:

(11) $$A_{postive}=\frac{\Delta C_{positive\max}}{C_{\max}-C_{\min}}\times 100\%=\frac{0.76}{50-8.33}\times 100\%=1.82\%,$$

(12) $$A_{negative}=\frac{\Delta C_{negative\max}}{C_{\max}-C_{\min}}\times 100\%=\frac{0.6}{50-8.33}\times 100\%=1.44\%,$$

## 5. Conclusions

In this paper, the fiber-optic curvature sensor with strip type sensitive region is taken as the research subject. Through studying its sensing mechanism and underwater application, the following conclusions are obtained:

1. The accuracy of the model of bending power relative loss and bending radius is verified by measuring the optical power loss in the sensitive region of the fiber, and the characteristics of positive and negative bending are explained effectively.
2. The sensor conditioning circuit can effectively amplify and filter the weak voltage signal, and can simultaneously measure the curvature of different bending degrees with a good application range.
3. After adding a second sensitive region on one fiber, the output voltage of the fiber curvature sensor will decrease while the sensitivity will decrease, but the linear measurement range remains basically unchanged.
4. When the sensor is applied underwater, its linear measurement range can be defined as 8.3–50 m^−1^, which is improved compared with the linear measurement range of 0–16.67 m^−1^ of the existing products.

## Figures and Tables

**Table 1:** Measuring standard cylindrical curvature data.

Bending Radius(mm)	Theoretical Curvature(m^−1^)	Forward Output Voltage (mV)	Measuring Curvature (mm)	Backward Output Voltage (mV)	Measuring Curvature (mm)
120	8.33	298	8.26	484	8.32
100	10.00	284	10.11	486	10.26
80	12.50	262	13.09	519	12.93
60	16.67	241	15.91	549	17.27
40	25.00	174	24.92	602	24.73
20	50.00	165	49.52	782	49.56
